# Testable Candidate Immune Correlates of Protection for Porcine Reproductive and Respiratory Syndrome Virus Vaccination

**DOI:** 10.3390/vaccines11030594

**Published:** 2023-03-05

**Authors:** Andrew R. Kick, Alicyn F. Grete, Elisa Crisci, Glen W. Almond, Tobias Käser

**Affiliations:** 1 Department of Population Health and Pathobiology, College of Veterinary Medicine, North Carolina State University, Raleigh, NC 27607, USA; 2 Department of Chemistry & Life Science, United States Military Academy, West Point, NY 10996, USA; 3 Institute of Immunology, Department of Pathobiology, University of Veterinary Medicine Vienna, 1210 Vienna, Austria

**Keywords:** vaccination, PRRSV, correlates of protection, humoral immunity, IgG, neutralizing antibodies, T cell, IFN-γ

## Abstract

Porcine reproductive and respiratory syndrome virus (PRRSV) is an on-going problem for the worldwide pig industry. Commercial and experimental vaccinations often demonstrate reduced pathology and improved growth performance; however, specific immune correlates of protection (CoP) for PRRSV vaccination have not been quantified or even definitively postulated: proposing CoP for evaluation during vaccination and challenge studies will benefit our collective efforts towards achieving protective immunity. Applying the breadth of work on human diseases and CoP to PRRSV research, we advocate four hypotheses for peer review and evaluation as appropriate testable CoP: (i) effective class-switching to systemic IgG and mucosal IgA neutralizing antibodies is required for protective immunity; (ii) vaccination should induce virus-specific peripheral blood CD4^+^ T-cell proliferation and IFN-γ production with central memory and effector memory phenotypes; cytotoxic T-lymphocytes (CTL) proliferation and IFN-γ production with a CCR7^-^ phenotype that should migrate to the lung; (iii) nursery, finishing, and adult pigs will have different CoP; (iv) neutralizing antibodies provide protection and are rather strain specific; T cells confer disease prevention/reduction and possess greater heterologous recognition. We believe proposing these four CoP for PRRSV can direct future vaccine design and improve vaccine candidate evaluation.

## 1. Correlates of Protective Immunity

What constitutes an effective vaccine? Sterilizing or protective immunity and eventual pathogen eradication are the goal, and vaccines have been highly successful in numerous diseases with only one natural host (i.e., smallpox and polio in humans). For viruses that infect multiple species (e.g., rabies), population immunity remains the goal and continued vaccination is a requirement to prevent disease outbreaks. Immunologists and vaccinologists describe the quantifiable characteristics of successful vaccination as immune correlates of protection (CoP). Over the past 60 years, Dr. Stanley A. Plotkin has greatly advanced human vaccine development, research, and understanding the immunological mechanisms that provide protection; these immunological mechanisms that provide protection from disease are referred to as CoP [1]. Plotkin further divides CoP into mechanistic (mCoP: those with a mechanistic cause of protection) and non-mechanistic (nCoP: predicts but does not cause protection) [2]. Through the course of numerous reviews and his textbook, Plotkin champions six principles [3] for CoP that are critical to examine and understand human diseases (quoted directly below).

i. Large challenge doses may overwhelm vaccine-induced immunity and confuse the identification of correlates,
ii. The mechanism of protection is not necessarily the mechanism of recovery from infection,
iii. Most vaccines available today act through antibodies; however, the immune system is redundant and may protect through multiple mechanisms (paraphrased),
iv. Memory induced by vaccination may be crucial to protection, particularly in long-incubation diseases,
v. Correlates may vary according to individual characteristics, such as age, gender, and major histocompatibility complex,
vi. It is important to define protection against what; what is the prevention objective (paraphrased).

One final principle Plotkin highlighted in his reviews is that the T-cell response is important for protection [4,5]; but thus far vaccine protection is largely quantified by neutralizing antibodies (NA) as described in point iii and most currently highlighted in Table 3.4 within his textbook [1]. We will apply these same principles to Porcine reproductive and respiratory syndrome virus (PRRSV).

## 2. The Problem of PRRSV

Despite available, stable, affordable vaccines, PRRSV is an on-going problem for the worldwide pig industry [6,7]. The two PRRSV species’ (type 1, commonly identified/referred as European, and type 2 as North American) genome homology is about 60% [8,9]; they rose to prominence and were identified at roughly the same time in the late 1980s, early 1990s [10]. Since that time, PRRSV-1 and PRRSV-2 have spread around the globe. The PRRSV has been a detrimental porcine health disease for over 30 years: PRRSV has ebbed and flowed between periods of significant loss during emergence of new high pathogenicity (HP) or high virulence strains and other periods of general prevention and mild losses [11]. Boehringer Ingelheim introduced the Ingelvac PRRS (strain VR-2332) modified live virus (MLV) vaccine in 1994; numerous other biological companies followed with type 1 and type 2 MLV or killed/component vaccines [6,7]. Additional experimental vaccine types are described in a recent review [12] to include a technique to attenuate PRRSV (codon pair deoptimization), which we believe holds future promise for autogenous vaccines. In general, commercially available PRRSV vaccines suffer from the same limitations: they protect well against the vaccine strain, but their protection against emerging PRRSV strains is limited [7,13]. The significant question is: Why does PRRSV remain a problem even with available vaccines?

One part of the problem is common—management practices. Implementation of multi-site production in the 1980–90s [14] enabled the rapid spread of PRRSV [15,16], continuous flow nurseries are more at risk for PRRSV outbreaks compared to single site production [17,18,19,20,21], and limited space allocation and proximity contributes to transmission of respiratory pathogens like PRRSV [22,23,24]. The transmission of PRRSV was thoroughly reviewed by Pileri and Mateu [25]. As with any economic decision, the relevance of efficiencies in management practices outweigh losses from disease. The PRRSV resides at the intersection of husbandry management, economics, veterinary care/animal well-being, anti-microbial resistance from administration combating secondary infections [26,27], and vaccinology: pork husbandry management practices have maximized economic efficiencies while balancing animal well-being and care; endemic diseases like PRRS have significant costs [28] (est. 2013, $664 million: loss, vaccination, medications, other costs), but these costs do not invalidate the efficiencies in the multi-site system. Regardless, vaccinations have eradicated or substantially reduced the burden of other porcine viral diseases: Pseudorabies virus, Porcine Parvovirus, Porcine circovirus 2 among others [29]. These diseases are effectively controlled by vaccines administered (or previously administered) at the herd-level to sows or sows and piglets. As discussed, 28 years of PRRSV vaccines have not achieved the same level of protective immunity [30].

Therefore, what characteristics of PRRSV make it different from the previously mentioned porcine viral diseases and help it evade effective control by vaccination? One characteristic is certainly its high mutation rate. The PRRSV is a positive stranded, enveloped RNA virus of the family Arteriviridae [8]. The RNA viruses have characteristic high mutation rates because most RNA polymerases do not possess proofreading mechanisms: RNA viruses have measured mutation rates of 10^−4^ mutations per nucleotide copied [31]. This high mutation rate leads to high genetic diversity resulting in “quasi-species” of PRRSV or “a cloud of diverse variants that are genetically linked through mutation” [31]. Beyond mutation, numerous studies have also supported possible MLV return to virulence [32,33,34] and recombination as a mechanism for PRRSV diversity [35,36,37] including recombination between vaccine and wild type strains [38] or two wild type strains.

The genetic diversity of PRRSV is well-documented and most published research examines the diversity of open reading frame 5 (ORF5), which is approximately 600 bp of the 15 kb PRRSV-2 genome [39,40]. The ORF5 codes PRRSV glycoprotein 5 (GP5), which has been implicated for host cell infection and as a target of NA [6]. The possibly outdated strain designation based upon the restriction fragment length polymorphism (RFLP) pattern [41,42,43,44] is being updated with whole genome analysis as sequencing technologies become more affordable and available [41,45]. Two recent studies evaluated strain diversity utilizing whole genome sequencing to compare genetic differences between lineage 1 strains with a 1-7-4 RFLP with previously circulating strains [42,45]. Genetic sequences can vary greatly between 1-7-4 strains and strain similarity does not necessarily correlate with pathogenicity [45]. This genetic diversity challenges veterinarians to constantly reevaluate the effectiveness of a chosen PRRSV vaccine for their farm. Future studies should aim to establish a method to timely assess PRRSV vaccine immunogenicity and use established CoP to predict the efficacy of available PRRSV vaccines against emerging field strains. This method would strongly facilitate the determination of the most effective vaccination strategy against newly emerging and circulating PRRSV strains.

The second characteristic providing challenges for vaccinologists and veterinarians is PRRSV’s immunosuppressive capacity. The PRRSV infects porcine macrophages expressing the scavenger receptor CD163 and possibly other cells from the monocytic lineages [8,46,47]. Impairment of lung macrophages in nursery pigs makes them susceptible to secondary bacterial infections, pneumonia, and other respiratory diseases [48], while PRRSV infection in pregnant sows can result in high litter mortality and abortions [49]. Butler et al. described in great detail some explanations for PRRSV evading vaccine-control: PRRSV has a rapid mutation rate and genetic diversity; identification of the critical viral epitopes for NA are limited [50]; and PRRSV has the ability to dysregulate the neonatal immune system [30]. In addition to investigating NA epitopes, there are on-going efforts to identify the largely unknown PRRSV T-cell epitopes [51,52,53]. With respect to immunosuppression, PRRSV research is extensive with the predominance of evidence implicating PRRSV suppression/inhibition of type I interferons [54,55,56]. Beyond type I interferons, Ma et al. reviewed the possible roles of PRRSV to suppress other innate immune defenses: cytokine production, tripartite motif proteins, microRNA, and small ubiquitin-related modifier E3 ligase activity [54]. Additionally, the impact of PRRSV killing lung macrophages cannot be neglected either in its role of immunosuppression or the vulnerability to secondary infections with porcine respiratory disease complex. Regarding the adaptive immune system, PRRSV has long been suspected of inhibiting thymus function through atrophy and thymocyte apoptosis and this is being substantiated with a growing body of evidence [57]. Less clear is whether PRRSV induces immunosuppression through early regulatory T cell (Treg) induction. Tregs are able to suppress the immune response [58,59] which can then lead to persistent infection [60]. The PRRSV research is conflicting with several studies supporting PRRSV Treg induction [61,62,63,64,65] while others did not find an active role [66] or were inconclusive [67]. Finally, when assessing blood transcriptional modules (BTMs) related to innate and adaptive immunity in the early immune response against two PRRSV-2 wild-type strains and a MLV, there was a fundamentally different immune response to the less immunogenic MLV than the robust response observed in the wild-type strains [68]. As we will observe in our review, PRRSV strains, vaccination/challenge timelines, and pig age all greatly affect accurately characterizing the immune response, which emphasizes our fundamental question: what immune CoP should PRRSV vaccination studies evaluate?

The third challenge for PRRSV control is its nature to infect and cause disease in pigs of different ages: PRRSV not only leads to reproductive issues in sows, but it also drives respiratory disease mainly in young animals. To protect sows from reproductive losses, commercial farms vaccinate selected gilts prior to breeding. Vaccination should result both in protection from PRRSV-related abortion during pregnancy and then provide maternal-derived immunity (NA and maternally transferred memory-cells) to piglets after farrowing and early nursery phase after weaning [30,69,70]. Multiple studies have confirmed transfer of maternally-derived antibodies (MDA) and memory T cells in colostrum to piglets with detectable NA titers [71,72,73,74,75,76], antigen reactivity [77,78], and protective effect [79,80]. While this maternal-derived immunity protects piglets, it also generates a challenge: to limit the detrimental effect of MDA, MLV vaccinations are usually administered in weaned pigs. As weaning is also the time of transfer of piglets from the farrowing house to a nursery farm, MLV PRRSV vaccination will be administered at the same time of a co-mingling event and exposure to circulating strains on the nursery farm. Based on the delay in a primary adaptive immune response, it is unlikely that this PRRSV vaccination at the same time as infection will provide protection. To provide more time for an adaptive immune response before entrance to a nursery farm, PRRSV vaccination would have to be administered early in life. However, in addition to the MDA, the less responsive nature of adaptive immune cells in young pigs [81] will provide a further barrier for PRRSV vaccines to generate robust immunity. That said, recent studies that evaluated the administration of a PRRSV MLV vaccine at 1-day of age showed promising results to overcome MDA and decrease viremia, viral shedding, and lung lesions [82,83,84,85]. These studies might provide a solution for the challenge to not only protect sows from reproductive issues but also young piglets from respiratory disease at a time of elevated stress as well as potentially exposure to high loads of PRRSV. In summary, PRRSV vaccinations are administered to gilts/sows during gestation, to young pigs upon transfer to their nursery stage, and study results on vaccinating newborn piglets are encouraging. These different ages for vaccination accentuate our point that age-based CoP are required as newborn, nursery, and adult pig immune responses are fundamentally different [81,86,87].

In summary, there are several factors that affect PRRSV vaccination efficacy: animal husbandry (multi-site production) allows for spread; PRRSV genetic diversity and rapid mutations result in quasi-species; the immunosuppressive nature of PRRSV limits vaccine efficacy; and vaccination timelines coincide with transfer from farrowing to nursery and often initial exposure to PRRSV. To overcome these obstacles, vaccines need to induce immunity to effectively protect the host. The big question is: what are the characteristics of effective PRRSV immunity? The clear definition of CoP not only provides the answer to this question, but it bears two additional advantages: first, vaccinologists can better design future vaccine candidates; and second, CoP can be used to timely and adequately predict the potential of vaccine candidates.

## 3. Plotkin’s Principles of Correlates of Protection Applied to PRRSV

The immune response to PRRSV can be subdivided into three main components: innate, antibody-mediated, and cell-mediated [51]. The innate immune response is critically important and has been reviewed extensively often in the context of PRRSV induced immune dysregulation or suppression [30,46,48,51,88,89]. However, while the innate immune response can develop a “de facto innate immune memory” (=trained immunity) [90], its role in and quantification for vaccine efficacy and CoP against PRRSV are still limited. Accordingly, we will evaluate PRRSV CoP in the context of the antibody- and T-cell-mediated immune response. We will organize our argument by first consolidating Plotkin’s principles into four hypotheses for the PRRSV CoP:

I. Effective class-switching to systemic IgG and mucosal IgA neutralizing antibodies is required for protective immunity.
II. Vaccination should induce virus-specific peripheral blood CD4^+^ T-cell proliferation and interferon gamma (IFN-γ) production with central memory and effector memory phenotypes; cytotoxic T-lymphocytes (CTL) proliferation and IFN-γ production with a CCR7^-^ phenotype that should migrate to the lung.
III. Pigs are vaccinated prior to breeding and at weaning: nursery, finishing, and adult pigs will have different CoP. Nursery pigs will generally have a higher neutralizing antibodies’ titer upon challenge than adult pigs: adult pigs should have a stronger IFN-γ response than nursery pigs.
IV. Neutralizing antibodies provide protection and are rather strain specific; T cells confer disease prevention/reduction and possess greater heterologous recognition.

In the following sections, we will discuss the PRRSV literature in detail regarding each of these four proposed PRRSV CoP.

### 3.1. Effective Class-Switching to Systemic IgG and Mucosal IgA Neutralizing Antibodies Is Required for Protective Immunity

Lopez and Osorio produced the seminal review on neutralizing antibodies (NA) and PRRSV immunity in 2004 [91]; this paper shaped the PRRSV communities’ understanding of the immune response to PRRSV and asserted the critical role of NA. Additional reviews have built upon that foundation with each concluding that NA are an important component of PRRSV protection [6,13,30,48,51,92].

To briefly describe the research and shifting evidence from those papers, initial PRRSV antibody research and opinions suggested that despite a strong serum anti-PRRSV IgG response within one week post infection, serum NA do not appear until generally 4 weeks post infection (wpi) or later [93,94,95]; or, if present earlier, specific IgG were not neutralizing the virus [93,96]. Equally troubling in the early discussion of NA and PRRSV was the dichotomy where serum NA presence coincided with viremia suggesting that serum NA are unable to clear viremia [91], and that concentration of protective serum NA are different based upon the age of pig [97,98]. Indeed, transfer of NA into pregnant females (titer of 1:16 by day 89 of gestation) prevented infection of the mother and offspring [97] and transfer of NA into weaned piglets at a 1:8 titer prevents viremia, but prevention in persistent replication sites (lungs and secondary lymph nodes) requires a higher titer [98].

Another scientist who has contributed significantly to our understanding of NA is the late Dr. Michael Murtaugh. Murtaugh’s recent papers from 2015 and 2018 demonstrated that sows from two PRRSV-exposed herds maintained high titers of NA to heterologous PRRSV-2 strains [99] and that passive transfer of NA into weaned pigs significantly reduced infection of PRRSV-1 and PRRSV-2 strains [100]. Not directly examined before in PRRSV, his lab also examined the role of interleukin 21 (IL-21) for B-cell proliferation and differentiation into antibody secreting plasma cells in the presence of PRRSV [101] and also the reactivity of B cells with PRRSV nsp7 [50,102]. The PRRSV research has almost exclusively focused on the product (antibody) and neglected the memory B cell (with the exception of J.E. Butler and M. Sinkora and now M. Rahe).

A survey of other recent PRRSV NA literature (since 2016) further supports their conclusions and our hypothesis that NA are a PRRSV CoP (summarized in Table 1 and described below). Every study is different, so we have organized the studies based upon the age of pig, vaccination type, and NA response. Before beginning our analysis, we must mention a debated topic in vaccine development for viruses infecting antigen-presenting cells: antibody-dependent enhancement (ADE). Studies across hosts and viruses confirm this phenomena that non- or sub-neutralizing antibodies can increase infection rates in animals by enhancing viral entry into cells they infect [103]. Within PRRSV research, Yoon et al. was the first to demonstrate ADE as a mechanism to increase infection with injection of sub-neutralizing amounts of IgG and identified the nucleocapsid and glycosylated envelope proteins as the targeted epitopes [96]. Over nearly three decades, additional mechanisms for PRRSV ADE have been gained to include the viral epitopes responsible: nucleocapsid protein, GP5 [104] and Fc receptors and mechanisms involved [105,106]. The GP5 is also identified as a viral epitope for NA [104] demonstrating the complexity in vaccine design and assessment for a virus where sub-neutralizing antibodies can increase infection. Nevertheless, as we will see in our NA literature survey, NA are achievable and critical for the immune response upon challenge and for viral clearance. To begin with MLV vaccinations in farrowing and nursery-age pigs, piglets vaccinated with PRRSV MLV at one day of age with [83] or without [107] PRRSV maternal antibodies showed better performance than unvaccinated animals. In a nursery-age pig study to investigate intramuscular vs. intradermal MLV PRRSV-1 vaccination and heterologous challenge, serum NA were detectable at 21–35 days post vaccination (dpv) and continued at equivalent levels through 35 days post challenge: both vaccination methods resulted in reduced viremia and symptoms in vaccinated animals [108]. With four different PRRSV-1 vaccines (killed virus and MLV), serum NA in nursery-age pigs were present 21 dpv and low levels of anti-PRRSV IgA and IgG were also generally present in the bronchoalveolar lavage (BAL) 14 dpv [109]. A heterologous DNA-vaccine in combination with MLV PRRSV-2 achieved serum NA in nursery-age pigs and improved clearance of HP virus upon challenge compared to unvaccinated pigs [110]. A study evaluating a MLV PRRSV-2 demonstrated significant improved performance in vaccinated nursery-age pigs upon heterologous challenge; however, serum NA titers were not detectable throughout the study [111]. In a separate MLV PRRSV-2 vaccination/challenge study in nursery-age pigs, serum NA titers were very low at 28 dpv; however, the serum NA rose rapidly upon heterologous and homologous challenge and were significantly higher than unvaccinated challenged pigs through 28 days post infection (dpi) [112]. Two PRRSV-2 strains attenuated by codon-pair deoptimization (CPD) were used as vaccines in nursery-age pigs compared to the infective wild-type counterparts, and achieved similar serum NA levels with significantly reduced lung lesions at 14 dpi; in a subsequent study, upon heterologous PRRSV challenge, the vaccinated groups exhibited reduction of all evaluated PRRSV infection criteria [113], which built upon the promising CPD attenuated vaccine results of two previous studies [114,115]. Finally, though serum NA were not examined in a PRRSV-2 vaccination/challenge study in nursery-age pigs, anti-PRRSV antibodies were detected following MLV-2 vaccination and upon challenge with a heterologous PRRSV-2 strain; as well, the vaccinated group had reduced viremia and duration of viremia compared to the unvaccinated group [116]. In summary, we observe MLV vaccination in nursery-age pigs results in low-level NA at 21–35 dpv. Nevertheless, despite these low levels, upon homologous or heterologous challenge, vaccinated pigs have reduced viremia, symptoms, and a much stronger NA response than unvaccinated pigs.

Vaccination studies of older pigs are less numerous, but upon MLV vaccination we often observe a complete protection against challenge. A 3-dose series of MLV PRRSV-1 in finishing pigs resulted in homologous serum NA titers at 21 dpv through 70 dpv: this vaccination resulted in sterilizing immunity in three of eight heterologous challenge groups [117]. In a novel vaccination study to better understand the B-cell response to PRRSV-2, Rahe et al. observed serum NA against two heterologous strains in finishing pigs at 118 dpv: these heterologous NA provided sterilizing immunity upon challenge at 118 dpv [50]. In a MLV PRRSV-2 vaccination and heterologous challenge study in gilts, serum NA titers were low prior to challenge (≤1:16 titer), but NA increased strongly after challenge and improved gilt reproductive performance [80]. Vaccination with a MLV PRRSV-2 vaccine that produced serum NA improved reproductive performance in gilts challenged with PRRSV-1 and PRRSV-2 strains [118]. Vaccination of sows prior to breeding with a MLV PRRSV-2 resulted in serum NA titers and significantly better reproductive performance than unvaccinated sows upon exposure to active circulating PRRSV strains [119]. In each of these studies, the MLV vaccinated pigs displayed a stronger NA response than unvaccinated pigs and were better protected upon heterologous challenge.

With respect to other types of vaccinations hoping to produce a more protective immune response than MLV vaccinations against heterologous challenge, the results are varied. In nursery-age pigs, a novel killed PRRSV-2 plus adjuvant intranasal vaccine induced serum NA detectable 14 dpv with a strong protective effect upon PRRSV-2 challenge 28 dpv and significantly higher NA than other vaccination combinations out to 35 dpv (7 dpc) [120]. A killed PRRSV-2 vaccination in nursery-age pigs resulted in serum NA 10 dpc for the homologous strain and decreased symptoms in vaccinated pigs [121]. A strain recovered from a cDNA clone of a PRRSV-2 isolate displayed homologous serum NA at 77 dpi; however, the antibodies did not neutralize 12 other PRRSV-2 isolates in-vitro [122]. A different engineered vaccine generated strong serum NA titers in nursery pigs; the vaccinated group had significantly better performance upon homologous challenge than the unvaccinated group; however, against a high pathogenicity PRRSV-2 heterologous strain the protection offered by vaccination was gone [123]. A chimeric PRRSV-2 vaccine in nursery-age pigs resulted in serum NA titers against two heterologous PRRSV-2 strains and upon challenge, the vaccinated pigs had reduced viremia and improved performance against those heterologous strains [124]. Additionally, three chimeric virus vaccinations combining different genetic combinations of two common PRRSV-2 strains did not produce broad NA at 42 dpi in nursery-age pigs; however, upon challenge with either heterologous virus, viremia was nearly undetectable in vaccinated pigs [125]. Finally, a commercially available PRRSV subunit vaccine (PRRSFREE, Reber Genetics Company, Taipei, Taiwan, Republic of China) that induced serum NA, protected gilts during PRRSV-1 and PRRSV-2 challenge, while all unvaccinated gilts aborted [126]. In conclusion, these vaccination and challenge studies confirm our thesis, serum NA are critical to preventing PRRSV infection as upon challenge a strong NA response correlates with reduced viremia, lung lesions, and PRRSV symptoms. In nursery pigs, though, we observe challenges in inducing heterologous protective immunity after vaccination.

One more theme concurrent through many of these studies is the narrow neutralizing capacity of NA: numerous papers report creative techniques for examining the heterologous reactivity of NA; yet, at the molecular level we remain challenged in predicting whether NA against a homologous strain will be cross-reactive against heterologous strains. In a novel study, Martinez-Lobo et al. attempted to evaluate the susceptibility of different PRRSV-1 strains to neutralization: strains were able to be categorized by their neutralization phenotypes, but this did not correlate with GP3, GP4, and GP5 epitope sequences [127]. Conclusively, genomic data are not adequate to predict a heterologous immune response. Therefore, immunological assays need to be applied to improve vaccine-induced cross-protection. Recently, progress was made in identifying MHC-I presented epitopes to CTLs [128]; however, based on the central role of the CD4^+^ T-cell response in heterologous protection [129], identifying MHC-II presented CD4^+^ T-cell epitopes need to be established to facilitate a research-based approach in designing more cross-protective PRRSV vaccines.

Our research team conducted three different animal trials to investigate the role of NA in vaccination, infection, and clearance for homologous and heterologous PRRSV-2 strains. First, unlike the paradigm of a significant delay between serum anti-PRRSV IgG, we observed serum NA to homologous virus with little delay from the serum anti-PRRSV IgG response in our infected groups with strains NC174 and NC134; serum NA levels rose by 14 dpi (NC174) and 7 dpi (NC134) and peaked at 28 dpi (NC174) and 42 dpi (NC134). These results were validated by fluorescent focus neutralization (FFN) that confirmed positive NA titers in 4/12 pigs by 7 dpi, and all animals had positive serum NA titers by 21 dpi [130]. In addition, the peak at 28 and 42 dpi coincided with drop of viremia to background levels in most pigs as well. Conversely, as is characteristic for a VR-2332 MLV inoculation and infection, homologous serum NA in the VR-2332 group were delayed becoming positive (in accordance with our flow cytometry based test) at 42 dpi and rose until the end of the study. Serum NA responses against heterologous viruses were different and not based upon ORF-5 similarity: the ORF-5 similarity between NC174 and NC134 was 86% compared to NC174 and VR-2332 (87.9%) or NC134 and VR-2332 (84.8%); the NC174 and NC134 infected pigs’ serum NA had strong cross-reactivity for both groups with 96% suppression by 35 dpi (NC134) and 49 dpi (NC174). Despite the ORF-5 similarities between the strains and a strong NC134-NC174 NA cross-reactivity, the VR-2332 MLV vaccinated pig serum NA exhibited no cross-reactivity with NC134 and limited cross-reactivity with NC174 [130].

When we evaluated the cross-reactivity of a new PRRSV-2 MLV vaccine, vaccination did not result in detectable serum NA at 28 dpv. However, upon heterologous challenge with one of four different PRRSV strains, only the vaccinated pigs developed serum NA titers against three of four strains at 14 dpc [129]. In the case of a killed autogenous NC174 vaccination administered to gilts after VR-2332 MLV vaccine administration, autogenous vaccination resulted in serum NA titers to the NC174 strain in gilts prior to farrowing, the offspring at weaning (2 weeks of age) had corresponding maternal-derived serum NA titers and upon challenge with the NC174 strain at weaning, viremia remained significantly lower than weaned piglets from gilts only vaccinated with the MLV: lung interstitial pneumonia histology scores were also lower in the NC174 maternally vaccinated group than the MLV-only group. Serum NA titers of the offspring dropped over the first 14 dpi/post-weaning and then remained constant until the completion of the study (28 dpi). Serum NA titers of the offspring from some of the MLV-only vaccinated gilts began to have detectable serum NA titers at 28 dpi; viremia and interstitial pneumonia histology scoring by 28 dpi was not different between groups. This study confirmed MDA to a homologous strain boost immunity in piglets [76].

Each of these studies examined serum anti-PRRSV IgG and/or NA: evaluating anti-PRRSV IgA in oral fluids has existed for almost 15 years but has recently been adapted to evaluate other routes of secretion/collection (nasal swabs and lung lavage). We briefly will summarize the available research on anti-PRRSV IgA. With respect to the role of secreted IgA in the humoral immune response and as a CoP, an intranasal vaccine induced anti-PRRSV IgA at 28 dpv and a generally stronger immune response resulting in improved performance upon challenge [120]. Differently, in our MLV study, vaccination did not result in detectable heterologous anti-PRRSV IgA in nasal swabs at 28 dpv. However, at 14 days after challenge with heterologous PPRSV strains, two vaccinated groups had higher IgA levels in BAL [129]. Additionally, we examined the presence of anti-PRRSV IgA in nasal swabs. We detected anti-PRRSV IgA by 7 dpi (NC134) or 10 dpi (NC174) in infected pigs with a peak at 14 dpi and detectable levels extending until 42 dpi (NC134) and 63 dpi (NC174). The VR-2332 MLV vaccinated piglets had a positive nasal swab IgA level by 14 dpi, but their levels were significantly lower compared to NC134 and NC174 infected pigs, they hovered around the limit of detection and varied based upon the individual animal response [130]. Other authors have suggested that anti-PRRSV nasal IgG are also an important CoP for the mucosal anti-PRRSV response induced by vaccination; they did not detect anti-PRRSV IgA [131]. Finally, in oral fluids, Ruggeri et al. found that anti-PRRSV IgA reduced virus replication and macrophage susceptibility to infection [132]. We believe vaccine induction of IgA in the respiratory tract is critical for protective immunity. At this point, the data is incomplete, but we assert PRRSV research will ultimately support our CoP as more research quantifies IgA’s role in infection prevention.

In summary, the last seven years of research investigating the role of serum NA in PRRSV protection have solidified that serum NA titers continue to play a critical role in reduction of viremia and improved performance for homologous and heterologous PRRSV challenge trials. The diversity of vaccines, age of pigs (nursery/sows and gestation), study design, and PRRSV challenge virus make drawing any specific required titer levels for protection not applicable: this fact is even more relevant based upon Plotkin’s first principle that “large challenge doses may overwhelm vaccine-induced immunity confusing CoP” [3]. In these studies, challenge doses generally range from a 50% tissue culture infectious dose (TCID_50_) of 10^5^ to 10^6^ administered to nursery-age pigs intranasally or intramuscularly; in studies with passive exposure to circulating PRRSV strains, MLV vaccinations can result in a marked reduction in viremia against the circulating heterologous strains [75,111,133]; Hermann et al. examined the probability of PRRSV infection and determined that intranasal exposure of 10^3^ resulted in 50% infection [134]. Perhaps, our persistence in high challenge doses overwhelms the IgG and IgA present in the respiratory tract allowing local PRRSV infection to occur and subsequent rapid viral expansion in the lungs; viremia, then, is not quickly sterilized in nursery-age pigs due to the susceptibility of macrophages to PRRSV infection and the barrier between circulating NA in the blood and PRRSV expansion in the lungs. Integrating a natural transmission model by contact with infected animals [75,135,136,137] is conducted infrequently as it presents synchronization and transmission failure risks, but it may be an approach to better predict vaccine efficacy. While PRRSV infections through wounded skin occur, most infections are transmitted via the oronasal route or semen. Particularly in developed countries and large-scale pig production, infections through semen can be prevented by solely using PRRSV-free semen for artificial insemination. Hence, based on its active transport through epithelial cells such as lung epithelial cells, IgA plays a major role in the protection of pigs against PRRSV. Accordingly, possibly a more accurate predictor of NA as a CoP is combining serum NA with anti-PRRSV IgA in the respiratory tract (nasal or oral fluids). Plotkin described this CoP for influenza because like PRRSV, influenza infects cells in the mucosa (epithelial cells not alveolar macrophages) [138]. He described the role of IgG and IgA as synergistic CoP with both being responsible for a significant drop in viral shedding compared to either individually.

In conclusion, PRRSV researchers have generally agreed that serum NA are an important measure of vaccine efficacy and a likely CoP, however, due to diversity of PRRSV strains and vaccines, differing anti-PRRSV immune responses between young and adult pigs, a specific protective serum NA titer has not been determined. In addition to serum NA, we believe effective evaluation of the ability of mucosal IgG and IgA to neutralize PRRSV prior to infection will be an important CoP and is the protection-causing mechanistic CoP (cause/effect) for what is observed in the relationship between infection, shedding, viremia, and serum NA.

### 3.2. Vaccination Should Induce Systemic CD4^+^ T-cell Memory and Lung CD8^+^ T-cell Memory

The anti-PRRSV cell-mediated immune (CMI) response lacks the depth and body of research of the humoral immune response. In general, the anti-PRRSV CMI response is described by IFN-γ (enzyme-linked immunosorbent spot) ELISPOT assay. As with serum NA, the scientific community describe the importance of the anti-PRRSV CMI response but in general agree that compared to human diseases researched in mice, the T-cell response to PRRSV is poorly understood. With respect to T-cell CoP, the systemic CD4^+^ T-cell response is so far the only T-cell response that has been proposed as a CoP for PRRSV [129]. This CoP has also been determined for other viral diseases: Plotkin concluded that the CD4^+^ T-cell response is critical for cytokine production to direct the immune response and antibody production with long-lasting protective memory as well as CTLs preventing infection/replication at sites of infection: “antibodies prevent infection whereas cellular responses control infection once replication has been established [3]”.

Most studies utilized IFN-γ ELISPOT assays either completely or partially as the measure of CMI. The IFN-γ ELISPOT assay is very sensitive with limited background IFN-γ^+^ cells and has been very useful in explaining the CMI response to PRRSV over the past 20 years; however, the IFN-γ ELISPOT assay is a limited tool for determining a CoP due to its lack of specificity of cell type since it is unable to differentiate the lymphocyte type or T-cell phenotype. An abbreviated summary of the IFN-γ ELISPOT assay data is described in Table 1. The T-cell gene expression [68,133,139] and cytokine production is also complementary [139,140,141,142] and especially critical for deciphering the role of Tregs [62,64,143,144] and innate immunity [46]; regardless, characterizing gene expression or cytokine production on their own is not specific enough to be a CoP for PRRSV. Differences in systemic or local T-cell populations following PRRSV infection or vaccination is also instructive and is a common method for describing the T-cell response: the anti-PRRSV response is inferred from changes in populations of T cells in peripheral blood, lungs, and lymph nodes. In general, after initial lymphocyte trapping, PRRSV infection increases the presence of T cells in the blood [67,145,146,147] and sites of infection following PRRSV infection [62,141,147,148,149,150] and specific increases in CD8α^+^ T cells in the lymph nodes [150,151] and lungs [67,150]. Population changes are indicative of effects of PRRSV vaccination; however, being able to quantify a specific anti-PRRSV CMI is required.

Here, we will describe the chronological progression of identifying specific proliferating and IFN-γ producing phenotypes upon PRRSV restimulation. The foundational paper by Bautista et al. provided the basis for future PRRSV-work that serves as the CMI CoP (characterizing specific T-cell phenotypes that proliferate and produce cytokines in response to PRRSV): following infection, lymphocyte proliferation from peripheral blood was detected 4 wpi, peaked at 7 wpi, and returned to background by 11 wpi in adult pigs [152]. Meier et al. built upon this foundation by identifying the T-cell subsets of IFN-γ^+^ cells in ELISPOT assay: >90% were CD4^+^CD8α^+^ memory T helper cells [153]. Costers et al. observed CTL proliferation in peripheral blood mononuclear cells (PBMCs) from PRRSV-infected nursery/finishing pigs upon PRRSV-1 restimulation increasing from 14 dpi to 56 dpi [154]. Using ELISPOT assay and flow cytometry combined, Ferrari et al. showed that vaccinated nursery pigs had higher CD8α^+^ IFN-γ^+^ cells than unvaccinated pigs 35 days after environmental exposure upon restimulation with the vaccine strain and a heterologous PRRSV isolate [155]. In the case of nursery pigs with either the MLV vaccine or heterologous challenge restimulation, at 21 dpc vaccinated challenged pigs had a higher proportion of CD4^+^CD8α^+^IFN-γ^+^ than unvaccinated unchallenged pigs [156]. Mair et al. then utilized violet proliferation dye to characterize the specific CD4^+^ T-cell response in nursery pigs to MLV PRRSV-2 restimulation and differentiated proliferating cells upon a naïve, central or effector memory phenotype observing a higher percentage of effector memory proliferation with adjuvant addition [157]. Sirisereewan et al. determined that vaccination (MLV or DNA-MLV PRRSV-2) and subsequent challenge in nursery-age pigs increased serum NA and CD3^+^ IFN-γ^+^ cells following vaccination and challenge, which reduced PRRSV viremia and symptoms but did not protect from a HP PRRSV-2 strain [110]. Cao et al. expanded upon T-cell phenotypes by analyzing the T-cell phenotypes (CD4^+^, CD8α^+^, CD4^+^CD8α^+^, and TCR-γδ T cells) for IFN-γ production and CD107a expression to characterize the CTL response to specific identified PRRSV-2 epitopes from adult pig blood samples [52]. Concurrently, Madapong et al. evaluated multiple PRRSV-1 and PRRSV-2 MLV vaccines in nursery-age pigs to 35 days post vaccination (dpv) followed by a 7 day challenge, assessing proliferation and IFN-γ production in the T-cell subsets with flow cytometry: proliferation was not detectable; IFN-γ production was highest in CD8α^+^ T cells upon homologous PRRSV restimulation [158]. In nursery-age pigs, a DNA GP5-mosaic resulted in increased IFN-γ mRNA production upon PRRSV-2 heterologous challenge at 35 dpv [159]. A killed PRRSV-1 and booster in nursery pigs induced IFN-γ^+^ Thelper, CTLs, and T memory cells at 2 wpv [160]. PRRSV-2 MLV vaccination in nursery-age pigs induced CD8^+^ IFN-γ^+^ during challenge with PRRSV-2 and swine influenza [161]. In a study to determine immunogenic CD8^+^ T-cell epitopes, the CTL response to nsp1a and nsp1b was detected in finishing pigs and sows at 16 and 21 dpc respectively [128]. In two separate PRRSV-2 vaccination and challenge studies in nursery-age pigs, CD4^+^ and CD8^+^ phenotypes were both involved in IFN-γ production and observed 28 dpv [120] or 10 dpc [121]. After MLV vaccination with and without adjuvant, Chaikhumwang et al. quantified IFN-γ using flow cytometry and found that upon homologous and heterologous challenge, CD4^+^IFN-γ^+^ T cells were significantly increased in adjuvant-vaccinated piglets, which aligned with decreased viremia and symptoms [120]. In summary, though these studies are varied, a few key conclusions can be drawn: the T-cell response upon vaccination includes IFN-γ production and proliferation in both CD4^+^ and CD8^+^ T cells and generally appears at 28 dpv while the T-cell response upon PRRSV challenge occurs as quickly as 7–10 dpi.

Our lab has performed two PRRSV vaccination and/or challenge trials with an exhaustive T-cell analysis. In the first trial, we utilized a vaccination strain and two field isolates to inoculate 4-week-old pigs and follow the course of infection and immunity for 9 weeks. We utilized multi-color flow cytometry with two different restimulation set-ups to evaluate three key parameters: isolated peripheral blood T-cell phenotype proliferation after homologous and heterologous virus in accordance with the previously discussed T-cell subsets with the additional marker for CCR7 expression (tissue or lymph node homing) and Foxp3 for Tregs; T-cell central memory populations residing in the tracheobronchial lymph nodes after clearance of viremia; and lastly IFN-γ, tumor necrosis factor alpha (TNF-α), and IL-2 production along with CCR7 expression in T-cell subsets [67]. At 28 dpi, the most evident characteristics of the T-cell response was that T helper cell proliferation coincided with clearance of viremia and T helper CCR7 expression indicated a shift towards an effector memory phenotype while maintaining a similar proportion of central memory T helper cells; and the same effect was observed in IFN-γ^+^ cells. Proliferating and IFN-γ^+^ CTLs expressed a tissue-homing phenotype in PRRSV-infected pigs, but these characteristics for CTLs were not present in MLV-vaccinated pigs. The TCR-γδ phenotypes also displayed interesting characteristics over the course of the infection; as well, the heterologous response revealed similarities in cross-reactivity between strains similar to those observed with the humoral response already discussed [130]: MLV infected pigs displayed the best in vitro cross reactivity to high-pathogenic (HP) virus; low-pathogenic (LP) pigs had the best in vitro cross-reactivity with HP virus; and HP pigs displayed in vitro cross-reactivity to MLV and LP virus at the height of infection. In cells isolated from the tracheobronchial lymph nodes at 9 wpi, the infected LP and HP treatment groups displayed strong T-cell subset proliferation and cross-reactivity with each other, but limited cross-reactivity to the MLV [67]. A subsequent study we conducted with the previously mentioned maternal vaccination study further defined the importance of T helper cells and CTLs in the anti-PRRSV response [76]. In this study, we only evaluated intracellular staining of IFN-γ in T-cell subsets, as well as B cells and NK cells for 4 wpi. Peripheral blood CD4^+^ T cells exhibited a lower percentage of effector memory in the IFN-γ component than in our previous study with a higher percentage residing in the central memory component. This difference might be explained by the age of the pigs in the studies: Pigs were two weeks younger in the later study with fewer effector memory cells. Nevertheless, necropsy of the lung revealed high IFN-γ production in CD4^+^ T cells. This characteristic was consistent with CTLs also having the highest percentage of IFN-γ^+^ cells of all lymphocytes isolated from the lung 4 wpi. Viremia decreased between 2 and 4 wpi despite no detectable serum NA in the pigs from the MLV vaccinated gilts again demonstrating the central role of cell-mediated immunity (CD4^+^ T cells and CTLs) in recovery from infection. Additionally, except for at weaning prior to infection, the highest percentage of IFN-γ^+^ lymphocytes in peripheral blood were CD4^+^ T cells followed by CTLs at 2 and 4 wpi. These results emphasize the power of flow cytometry over IFN-γ ELISPOT assay: At 0 wpi, we observed CD21α^+^ cells as the highest percentage of IFN-γ^+^ cells and their representation at 2 and 4 wpi was replaced by CD4^+^ T cells and CTLs; with IFN-γ ELISPOT assay alone we would not have that lymphocyte fidelity. Using a similar flow cytometry approach, Proctor et al. studied both vaccine immunogenicity and efficacy of a MLV vaccine against four different PRRSV strains. Besides demonstrating heterologous efficacy of the MLV vaccine, this study performed correlation analyses between different immune parameters and vaccine efficacy parameters. Immune parameters encompassed lung IgA, serum IgG, and the differentiation, IFN-γ production and proliferation of systemic CD4^+^, CD8^+^, and TCR-γδ T cells. Efficacy parameters included were gross pathology, shedding, and viremia. Generally, while CoP could be detected against all efficacy parameters, predictions best predicted the effect of the immune responses on viremia. From an immune perspective, both the humoral and the systemic CD4^+^ T-cell response were the best CoP for heterologous protection against PRRSV. However, only the CD4^+^ T-cell response was analyzed strain-specifically [129].

In conclusion, actual studies evaluating the specific contribution of T-cell subsets and phenotypes are yet limited; but with the advancement of the flow cytometry antibody repertoire for swine, flow cytometry will enable a much higher-level of fidelity to characterize the CMI response to PRRSV. Accordingly, we predict that effective vaccines should induce systemic CD4^+^ T-cell memory and lung CD8^+^ T-cell memory. In flow cytometry, we should attempt to better understand the below measures:

1. Vaccination should induce virus-specific peripheral blood CD4^+^ T-cell proliferation and IFN-γ production with central memory and effector memory phenotypes.
2. Proliferating and IFN-γ producing CTLs should indicate a CCR7^-^ phenotype that allows them to migrate to the site of infection—the lung or the reproductive tract.

### 3.3. Nursery Pigs Will Generally Have a Higher NA Titer upon Challenge Than Adult Pigs: Adult Pigs Should Have a Stronger IFN-γ Response Than Nursery Pigs

Over the past 30 years, Dr. Armin Saalmüller’s institute has contributed significantly to our understanding of swine immunology and developed crucial tools to evaluate the immune response. A sampling of their work illustrates the differing perspectives and analysis required for determining PRRSV CoP in nursery, finishing and adult pigs. The T helper phenotypes in peripheral blood change dramatically between newborn, nursery, and adult pigs with a continuous shift from naïve (CD3^+^CD4^+^CD8α^-^CCR7^+^(CD27^+^)) to central memory (CD3^+^CD4^+^CD8α^+^CCR7^+^(CD27^+^)) to effector memory populations (CD3^+^CD4^+^CD8α^+^CCR7^-^(CD27^-^)) [86,162] as well as differences in other relevant phenotypic markers [86,87,163,164]. The IFN-γ production as a CoP is also age-dependent with adult pigs being better producers of various cytokines such as IFN-γ than nursery pigs [81]. Additionally, nursery-age pigs are more susceptible to prolonged PRRSV infection than the adult [30]. Accordingly, decreased IFN-γ production in nursery pigs could be a factor contributing to the longer duration of infection in nursery pigs.

With respect to humoral immunity CoP, MLV vaccinations can generate homologous and heterologous serum NA as soon as 21 days post vaccination that provide beneficial protection during homologous challenge or environmental exposure in gilts/sows [75,76,80,107,118,119,165]. For nursery-age pigs (taking into account vaccine diversity and the number of studies), our previous discussion in Section 3.1 highlights greater variability in NA induction with multiple studies reporting NA not detectable following vaccination and lack of cross-reactivity between strains for vaccine generated NA. One other point is the trend of strain VR-2332 to result in delayed NA consistent in vaccination and infection studies highlighted from Table 1 [111,130,153,161,165,166,167,168]; regardless, vaccine efficacy is attributable to VR-2332 as serum NA are detectable within 7–14 dpi after challenge. In our own NA study, we observed a considerable delay in generation of NA with a positive result occurring at 42 dpi, which increased through the end of the study (63 dpi) [130].

With respect to vaccination, the CoP will be different between vaccinated gilts/sows. Current vaccinations significantly improve reproductive performance, especially upon homologous or environmental challenge [75,76,118,119,169]. Maternally derived immunity (MDI) improves offspring performance upon PRRSV exposure during the nursery phase, though it is not completely protective [76,79,170]. If upon transfer to nursery phase, pigs are vaccinated and also are exposed to circulating strains, then it is unlikely maternal PRRSV vaccines will provide protective immunity to nursery-age pigs. Vaccination of newborn piglets [83,84,85] appears to be a strategy to overcome concurrent nursery vaccination and exposure; however, the vaccine’s effect must overcome MDA, the piglet’s immature immune system, and the 28 days or more [67] required for a maximal immune response. In the above studies, challenge occurred at 9 or 18 weeks after vaccination. To summarize, the CoP in nursery-age pigs must be defined by both MDI and vaccine generated immunity. These are two distinct categories and must be investigated accordingly.

Without at this point specifying exact NA titers or IFN-γ^+^ T-cell numbers, surveying the literature, we can postulate that serum NA titers in adult pigs at vaccination or even upon challenge generally remain relatively low (≤1:16) [76,80,118,119,165,167,169]; conversely, in young pigs the vaccination NA titers are often undetectable or low (≤1:8) but upon challenge or infection, NA titers can be much higher than the adult pig range and reach the assay maximum (1:512) [50,83,108,117,120,130,142,171]. With those titer ranges in view, we must also keep in mind that development of NA are PRRSV strain dependent and also vary greatly per individual animal. Because quantifying IFN-γ production in T-cell phenotypes is less standardized than NA titer quantification, we will only compare IFN-γ production results from our lab in isolated peripheral blood mononuclear cells taking into account the different antigen restimulation. In a *Chlamydia suis* vaccination study, we observed vaccinated gilts with a significant response at approximately 0.5% of CD4^+^ T cells upon antigen restimulation [172]. In our two challenge studies in nursery pigs, we observed the strong CD4^+^ T-cell responders to be closer to 0.25% IFN-γ^+^ upon antigen restimulation [67,129] with the exception of one group that responded close to 0.5% [129]. As more labs conduct flow cytometric analysis of T-cell IFN-γ production, the results will become more consistent, but we are confident that the adult pig IFN-γ response will be more robust than young pigs [81].

In conclusion, PRRSV vaccine CoP will be different in weaned/unvaccinated pigs, weaned/vaccinated pigs, and breeding gilts/sows. The maturity of the animal’s immune system will result in a more measurable response for IFN-γ production, NA titers, and T-cell phenotypes. Understanding the age-dependent changes of T-cell phenotypes/cytokine production and NA are critical for correctly interpreting and predicting PRRSV immune CoP.

### 3.4. Neutralizing Antibodies Provide Protection and Are Strain Specific; T cells Confer Disease Prevention/Reduction and Possess Greater Heterologous Recognition

Our final hypothesis can be observed with Table 1 as we compare the reported timeline for appearance of IFN-γ secreting cells (SCs) with serum NA. Table 1 illustrates the increased emphasis on identifying the characteristics of a heterologous response, as over the past seven years, the volume of research investigating vaccination with homologous and heterologous challenge is extensive emphasizing the challenges faced in production. A thorough study that emphasizes our overall hypothesis is Correas et al.: a novel vaccine/virus was administered and then IFN-γ SC numbers were compared for antigen recall of eleven PRRSV isolates at 63 and 77 dpi; IFN-γ SCs numbers were similar across PRRSV-2 isolates; however, serum NA were superior for the homologous strain with limited heterologous titers [122]. This relationship is repeated with multiple studies in Table 1. For example, Park et al. observed the IFN-γ response was generally heterologous while the NA response was homologous [113]. Nevertheless, even more important than understanding the homologous vs. heterologous response for this CoP is the clear relationship between vaccine-generated cell-mediated immunity through CD4^+^ memory cells and effective class-switching and serum NA upon PRRSV challenge. Balasch et al. is the perfect example of this principle: serum NA did not appear until 56 dpv but were maintained out to 125 dpv with similar IFN-γ SCs results; upon challenge, there was a 3-fold increase in the serum NA titer at 10 dpi in vaccinated pigs (IFN-γ SCs changes depended upon the vaccination) [83]. Numerous other studies reflect this relationship of a limited NA response upon vaccination, but rapid increase in NA upon infection [117,129,165,166,168,171]. On the other hand, this is not a uniform response as evidenced by Zuckermann et al. where the MLV vaccination was effective and protected pigs upon challenge from viremia and symptoms: in that case, serum NA did not rise upon challenge while for the other treatment group with an ineffective killed vaccine, serum NA rose rapidly along with viremia upon challenge infection [173]. This study perfectly illustrates this CoP: an ideal PRRSV vaccine will produce serum and mucosal NA that prevent PRRSV infection; however, if vaccination does not produce sterilizing NA titers, then the CMI primed by vaccination results in rapid antibody seroconversion and affinity maturation in B cells to produce NA that reduce viremia as well as memory T helper cells and CTLs that migrate to the cites of infection.

Our first study involved challenge only without prior vaccination of the infected animals; accordingly, T-cell and B-cell memory populations were developing simultaneously and CMI supported clearance of viremia and protection from follow-on infection. Briefly, as discussed in Section 3.1 and Section 3.2, we observed this principle in both of our fore mentioned studies: T cells displayed greater and different cross-reactivity to heterologous strains in ex vivo restimulation experiments than was observed for serum NA [67,130]. In our second maternal vaccination study [76], MLV (VR-2332) vaccinated gilts did not produce serum NA to the NC174 strain but did have a NA titer for VR-2332. The MLV + NC174 vaccinated gilts produced serum NA titers to NC174 and VR-2332. With respect to CMI, there was no difference in the T-cell IFN-γ response with NC174 restimulation between treatments reemphasizing the first tenet of this CoP: serum NA are generally strain specific while T-cell responses exhibit greater cross-reactivity. Secondly in this study, maternally transferred antibodies provided greater protection to the NC174 vaccination groups 14 dpi; however, by 28 dpi, the CMI response and reduction in viremia between all treatments was equivalent demonstrating the second tenet of this CoP: T cells do not prevent infection but reduce viral proliferation and disease.

## 4. New Approaches for Immune Correlates of Protection Evaluation

Besides briefly identifying the difficulties in vaccination of nursery-age pigs, our primary emphasis in this review was to first use Plotkin’s six principles for postulating four hypothetical CoP for PRRSV. Then we performed a survey of current PRRSV research to test if this research supports or falsifies our hypotheses. Our main findings regarding our four hypothetical CoP for PRRSV can be summarized as follows:

I. Serum NA either after vaccination or challenge correlate with reduced viremia and PRRSV pathology.
II. Cell-mediated immunity is quantifiable with IFN-γ production in specific T-cell phenotypes, and as expected in viral infection: induction of the T-cell response (T helper and CTL) results in clearance of infection and improved performance.
III. Age greatly affects the measured response to PRRSV: nursery-age pigs and gilts/sows demonstrate different characteristics in their immune response.
IV. Serum NAs induced from vaccination or infection are more likely to be strain specific than T cells, which enable an improved response across PRRSV strains.

Therefore, we believe this review determines that the literature confirms our four hypotheses.

Hence, we recommend the following direction for future PRRSV research based upon our four hypotheses:

1. Sustain evaluation of serum NA against relevant homologous and heterologous virus strains while developing new methods for evaluating the neutralizing capability of mucosal IgG and IgA. Unlocking our understanding of secreted IgG and IgA is the key to vaccines providing sterilizing immunity. Also, mucosal (intranasal) vaccines may be more likely to generate secreted IgG and IgA than intramuscular vaccinations as evidenced with other swine diseases [120,148,174,175,176,177]. Finally, assessing both inhibition of viral replication and viral transmission can further improve the testing of vaccine candidates.

2. The PRRSV immunology research should evaluate the CMI response with more specific methods than IFN-γ ELISPOT assay: gene expression, flow cytometry for specific T-cell phenotypes (memory, transcription factors, proliferation, cytokine production), and immunohistochemistry for T-cell resident memory populations are available tools to improve our understanding. Once our CoP are fully established, it should be possible to isolate circulating PRRSV strains, codon pair deoptimize the strains [176], and test the strains to demonstrate low or no viremia and a virus-specific IFN-γ producing T helper central and effector memory population in the blood with IFN-γ producing CTLs migrating to the lungs and periphery.

3. Gilt and sow vaccination provide improved immune protection from abortion during gestation against current circulating PRRSV strains and NA are transferred to suckling piglets. Little is known for PRRSV on the ability of passive transfer to cross into the mucosa to protect weaned piglets in the nursery. As Butler emphasizes, the neonatal pig is the most vulnerable to PRRSV in the period that maternal antibodies are decreasing before the weaning age (or earlier) and until PRRSV vaccination provides protection. Pigs are vulnerable during this time and so long as PRRSV is circulating in herds, this window of time remains a challenge. A novel weaning study that evaluates transfer and vaccine induced production of not only NA but also B-cell and T-cell memory cells would help our visualization of how to improve maternal vaccinations to protect farrowing and nursery-age pigs.

4. This CoP is the current industry model: MLV vaccines applied at nursery age improve performance, but do not provide protective immunity to the plethora of PRRSV strains circulating in the field. Biological companies, the reviewed research, and producers generally agree that the current line of vaccines does not completely prevent heterologous viremia, PRRSV clinical symptoms, lung lesions, or shedding; however, it does reduce the effects of PRRSV and significantly improve performance over unvaccinated animals. This is probably caused by the robust and flexible CMI response. The research demonstrates greater recognition of heterologous PRRSV strains in the CMI response than is inherent in serum NAs. The cell-mediated immune response enables recovery from disease caused by mainly heterologous PRRSV strains that was not prevented by the humoral defenses.

With respect to vaccination strategies, targeting the areas of greatest vulnerability (gestation and nursery) must remain the objective. Accordingly, ensuring a robust vaccination schedule during gestation with vaccines with the greatest demonstrated success ensures that fetuses are most protected from abortion and that MDI provides a barrier against strains circulating in farrowing. Second, continued research towards vaccines administered within the first 1–3 days after birth will provide the adaptive immune system time to develop protective immunity towards strains circulating in nursery barns. Ideally and practically, we believe this initial vaccine could be an intranasal vaccine generating a stronger mucosal response while gestational vaccines could remain intramuscular due to the different nature of PRRSV infection during gestation. Furthermore, in mice, mucosal vaccines could overcome maternal-derived immunity [178]; hence, early-in life mucosal vaccination might present a promising strategy to establish immunity against PRRSV at weaning.

In conclusion, the purpose of this review was to examine current PRRSV adaptive immune research from a new perspective by nominating four CoP for PRRSV vaccination. Of the CoP, the first two are measurable and can potentially be used as quantifiable measures for vaccine evaluation in the future: PRRSV neutralizing serum IgG, secreted neutralizing IgG and IgA titers, and a descriptive CMI response analysis. The third and fourth CoP are hypotheses to bear in mind when evaluating the first two hypotheses. The immune CoP for vaccination of nursery pigs should be different than breeding gilts/sows. Protective NA titers may be higher in nursery pigs and the proportion of effector memory and IFN-γ production achieved will be higher in gilts/sows: young pigs will have different measured values than adult pigs, yet both can be protected. If the humoral immune response (IgG and IgA) does not provide protective immunity, vaccination should result in a sufficient CMI response to generate central and effector memory cells to defeat infection and disease. As Plotkin stated in his latest review, “a biological fact is of great importance: that the immune system is redundant, and that more than one response may be a CoP” [5]. Our hope in this review is that the reader will have a broadened perspective on the amazing complexity and redundancy of the immune system as we work together towards vaccine-generated protective immunity and eradication of PRRSV.

**Table 1:** Comparison of pig age, IFN-γ secreting cells, and serum NA in PRRSV vaccination or challenge.

Strain	Pig Age	Vaccination	IFN-γ Response	Serum NA	Challenge	Viremia/Symptoms	IFN-γ Response	Serum NA	Reference
PRRSV-2	N	IN KV-2 +adj on KV-2	KV-2 + adj INC at 28 dpv	KV-2 + adj INC at 28 dpv (also nasal IgA)	HO WT-2 at 28 dpv	KV-2 + adj (Lung WT-2 DEC)	All vacc INC in CD4^+^, CD8^+^ phenotypes	All vacc INC; Adj-vacc INC highest	[120]
PRRSV-2	N	KV-2	NE	NE	HO WT-2	Vacc DEC, 7–10 dpc	Vacc INC 10 dpc in CD4^+^, and CD8^+^	Titer 10 dpc in Vacc	[121]
PRRSV-2	N	IM MLV-2	ND	ND (anti-PRRSV nasal IgA induced for vacc)	HE WT-2 28 dpv	DEC in Vacc	Vacc/unvacc HE INC 14 dpc; Vacc CD4^+^ and CD8^+^ INC over unvacc for NC174 & 1-4-2	INC in Vacc against ¾ strains (not NC174)	[129]
PRRSV-2	N	IM and ID MLV-2 and ID or IM PCV2	HO and HE SCs INC in vacc at 28 dpv	NE	HE WT-2	DEC in all vacc	HO & HE SCs in INC in vacc 7 dpc; unvacc detectable 7 dpc	NE	[179]
PRRSV-1	G/S	MLV-1 and second KV-1 3w before farrow	NE	INC in double Vacc and their piglets as measured weaning	CSE on two farms	Double vacc offspring DEC PRRSV+ and lower mortality	NE	NE	[75]
PRRSV-2	N	N/A	N/A	N/A	IN WT-2 or IM MLV-2	All viremic	HO and HE in WT at 28 dpi	HO at 14 or 21 dpi in WT and HE at 28 or 42 dpi in WT; MLV HO only and low	[67,130]
PRRSV-2	N, G	G series of MLV-2 and KV-2 during gestation	CD4^+^ largest IFN-γ producers 7d before farrowing	MLV-2 + KV-2 had WT NA; MLV only not HE	N, HO or HE WT-2 at 14d of age	Lung lesions DEC in MLV-2 + KV-2	No diff between vacc; IFN-γ prod shifts with age	MD NA detectable and prot in MLV-2 + KV-2	[76]
PRRSV-1	N	MLV-1 at 1 day-old	NE	NE	HE WT-1 at 28 dpv	DEC in VAC	INC SCs in Vacc and positively correlated with DEC viremia	NE	[85]
PRRSV-1	N	MLV-1 in conjunction with swine influenza A	NE	NE	HE WT-1 at 28 dpv	Co-infection at vacc did not affect viremia/symptoms	INC SCs in vacc at 15 dpc	Detected at 15dpc across challenged	[180]
PRRSV-1	F	IM MLV-1 (3 dose series); two strains	NE	HO beginning at 21 dpv through 70 dpv (3^rd^ vacc)	HE WT-1 various strains at 70 dpv	No clinical signs; vacc lower viremia	NE	Vacc with broadly NA achieved sterilizing immunity in 5/8 chall groups	[117]
PRRSV-2	N	IM MLV-2	ND	ND	HE WT-2 at 33 dpv with swine influenza	DEC in VAC, unless co-infected	Vacc induced CD8 prod: no difference post challenge	ND	[161]
PRRSV-1	N	IM KV-1 + adj + booster	Vacc induced Th, CTLs, Tm 2 wp booster in two trts	NE	HO WT-1 at 50 dpv	No sig diff between vacc/unvacc trts	Unvacc INC in Th, CTLs, Tm post challenge	NE	[160]
PRRSV-2	N	DNA GP5-mosaic or GP5-WT (IM & ID)	mRNA INC HO and HE at 35 dpv for GP5-mosaic; HO for WT	HO and HE at 35 dpv for GP5-mosaic; HO for WT	WT-2 IN and IM at 35 dpv with HO or HE strain	GP5-mosaic DEC viremia and pathology for HO and HE	NE	NE	[159]
PRRSV-1 PRRSV-2	N	MLV-1 (IM v. ID)	HO at 28–35 dpv	HO and HE at 21–35 dpv	HE WT-2, WT-1	Vacc DEC and ID vacc had lower viremia than IM	HE SCs appear in vacc 7 dpc	HO and HE continues through 35 dpc	[108]
PRRSV-2	F	IM MLV-2	NE	118 dpv for 2/3 HE strains (not 1-7-4)	HE WT-2	Viremia only detected in 1-7-4 challenged	NE	Vacc induced HE NA after challenge	[50]
PRRSV-2	G	MLV-2	Vacc INC SCs 42–135 dpv	Vacc INC 42–135 dpv	HE WT-2	Vacc INC reproductive performance	Vacc INC SCs	Vacc INC NA after challenge	[80]
PRRSV-2	N	N/A	N/A	N/A	WT-2	Peak at 10 dpc	IFN-γ+ cells early in local response peak at 21 dpc	Appeared 28 dpc	[150]
PRRSV-2	N	CPD of WT	HO/HE 28 dpv	HO only at 28 dpv	CPD vs. HE WT	CPD lower	HO/HE 14 dpi	HO only 14 dpi	[113]
PRRSV-1 PRRSV-2	mice	KV w/mAb adj in mice	HE at 28 dpv	mAb cross-reactive	N/A	N/A	N/A	N/A	[181]
PRRSV-1 PRRSV-2	G/S	MLV-1 or MLV-2	2 HE w/HO higher; 1 HO only 21 dpv	Both HE/w HO higher 21 dpv	Both HE WT-1&2	MLV-2 reduced 1&2	HE w/HO higher; 2 more SCs	HE w/HO higher	[118]
PRRSV-1	N	MLV or MLV w/DNA	MLV w/DNA higher SCs to peptides 13 dpv	NE	N/A	N/A	N/A	N/A	[131]
PRRSV-1	N	4 types (2 KV, 2 MLV)	Positive for all vaccines 21 dpv	MLV higher 21 dpv	HE WT-1	Virus shed lower in vacc	Positive to include unvacc/challenged	Inactivated higher 21 dpv	[109]
PRRSV-1	N,F	MLV IN or IM	SCs INC thru 125 dpv	Appeared 56 dpv thru 125 dpv	HE WT-1	Reduced in vacc	IN INC post-chall; IM DEC 10 dpi	3-fold INC vacc 10 dpi	[83]
PRRSV-1 PRRSV-2	G/S	MLV-1 & MLV-2	HE 21 dpv	HE 21 dpv	Both HE WT-1&2	Vacc higher RP	SCs higher 7 & 21 dpi	Titer higher 7 & 21 dpi	[169]
PRRSV-2	G/S	MLV-2	HO 70 dpv	HO 70 dpv	CSE	Vacc no viremia/higher RP	DEC at farrow	DEC at farrow	[119]
PRRSV-2	N	N/A	N/A	N/A	cDNA clone, FL12	N/A	SCs highest 63 & 77 dpi; genetic similarity did not affect SC with type 2 PRRSV	HO NA titers at 63 & 77 dpi; limited HE titers	[122]
PRRSV-2	N	MLV-2	Peaked 21 dpv	ND	HE WT-2 isolates	Vacc DEC	Rose rapidly thru 14 dpi	ND	[111]
PRRSV-1 PRRSV-2	N	WT-1 or WT-2	Low-level HO & HE post-chall	ND	WT-1 or WT-2 (HO or HE)	HO second chall; DEC	HO second chall; rapid rise 3 dpc	HO second chall; rapid rise 7 dpc	[171]
PRRSV-1	N	MLV-1	NE	NE	CSE	Vacc INC morbidity & survival	Vacc INC 7 wpv thru 16 wpv; unvacc higher Treg activation	NE	[133]
PRRSV-2	N	MLV-2 or MLV-2 + adj	Higher SC 14 dpv MLV + adj	NE	N/A	N/A	N/A	N/A	[157]
PRRSV-1 PRRSV-2	N	MLV-2	NE	NE	HE WT-1	Vacc reduced	Higher vacc SC 7–21 dpc	NE	[182]
PRRSV-1 PRRSV-2	G/S	MLV-2	SCs INC to 84 dpv; DEC 121 dpv HO virus	ND	HE WT-1 or WT-2	HO chall prot; HE chall semi-prot	SC to HO virus in vacc highest; all HO SC INC after infection	HO chall highest NA; reduced for HE chall	[165]
PRRSV-2	N	MLV-2	NE	ND	HO or HE WT-2	MLV + HO-2 chall prot	MLV + WT challenge had highest SCs	HO present 14 dpi	[166]
PRRSV-2	N	MLV-2	SCs INC 7, 14 dpv	ND	HE WT-2	Vacc DEC	SCs INC rapidly 7–14 dpc in vacc	ND	[156]
PRRSV-1	N	MLV-1 (IM vs. ID vs. adj only)	SCs to HO & HE at 21, 35 dpv	ND	CSE	Similar across trts	SCs INC to HO & HE at 35 dpi in vacc	Titers present after CSE in vacc	[155]
PRRSV-1	N	MLV-1, PCV2 or both	SCs INC after MLV-1	NE	CSE	Similar across trts	SCs INC all trts after CSE	NE	[183]
PRRSV-1	N	2x WT-1 isolates	WT-1(1) more SCs than WT-1(2)	WT-1(2) more NA than WT-1(1)	Challenge w/either WT-1 isolate	WT-1(2) no viremia; WT-1(1) low viremia	WT-1(1) more SCs than WT-1(2)	WT-1(1) more NA than WT-1(2)	[142]
PRRSV-2	N	N/A	N/A	N/A	WT-2	Viremic 7 dpi; 50% 56 dpi	Detected 28 dpi; peaked 42 dpi; steady 56–193 dpi	NA appear 42 dpi; peak 70 dpi; remain 193 dpi	[167]
PRRSV-1	G/S	MLV-1, KV-1	SCs background for both	ND	HE WT-1	KV viremic; MLV prot	Low, more SCs in MLV than background	KV rose 10 dpi: MLV background	[173]
PRRSV-1	F	N/A	N/A	N/A	WT-1	Clear w/in 7–14 dpi	SCs INC 14–70 dpi	NA appear 56 dpi in 60%	[145]
PRRSV-2	N	MLV-2	SCs peaked at 28 dpv, then DEC	NE	HE WT-2 cocktail	WT-2 persists in lymph nodes 67 dpi	No correlation SCs with virus presence in tissues 19 or 67 dpi	NE	[184]
PRRSV-2	N, F	Recombi-nant component in BCG	PRRSVSCs ND	Detectable 60 dpi	HO WT-2	Vacc DEC	PRRSV SCs ND	NA not INC 7 dpi	[185]
PRRSV-2	F	MLV-2 + adj	SCs peaked 4 wpi	Minimal thru 8 wpv	HE WT-2	Vacc lower/no viremia at 4, 7 dpc	SCs INC after chall thru 14 dpc	Vacc NA INC 14 dpc to vacc & chall	[168]
PRRSV-2	F	MLV-2, KV-2, PRV, or MLV-2 + adj	MLV-2 vac SCs INC thru 8 wpv; less than PRV	NA detectable at 8 wpv; less than PRV	N/A	N/A	N/A	N/A	[153]
PRRSV-2	N-A	N/A	N/A	N/A	WT-2	Persisted 3 wpi	SCs maintained 5–12 mpi	NA appear with SCs; DEC post-viremia	[186]

Table Abbreviations: N—Nursery (2 weeks+), A—adult, F—finishing (8 weeks+), G/S—gilt/sow, CPD—Codon-pair deoptimization, HO—homologous, HE—heterologous, KV—killed virus, dp—days post, wp—weeks post, v—vaccination, c—challenge, i—infection, 1—type 1 PRRSV, 2—type 2 PRRSV, MLV—modified live virus, WT—wild type, SC—secreting cells, NA—neutralizing antibodies, CSE—circulating strains exposure, N/A—not applicable, NE—Not evaluated, ND—Not detected, adj—adjuvant, vacc—vaccinated treatments, RP—reproductive performance, IN—intranasal, IM—intramuscular, ID—intradermal, PCV2—porcine circo virus 2, PRV—pseudorabies virus, INC—increased, DEC—decreased, prot—protected, BCG—*Mycobacterium bovis* Bacille Calmette-Guérin.

## Data Availability

Not applicable.

## Text Abbreviations

Porcine Reproductive and Respiratory Syndrome Virus (PRRSV); immune correlates of protection (CoP); cytotoxic T-lymphocytes (CTL); interferon gamma (IFN-γ); neutralizing antibodies (NA); high pathogenicity (HP); Modified Live Virus (MLV): Open Reading Frame 5 (ORF5): restriction fragment length polymorphism (RFLP): T-regulatory cell (Treg): blood transcriptional modules (BTMs); maternally-derived antibodies (MDA); immunoglobulin G (IgG); C-C chemokine receptor type 7 negative (CCR7-); weeks post infection (wpi); interleukin 21 (IL-21); antibody-dependent enhancement (ADE); glycoprotein 5 (GP5); bronchoalveolar lavage (BAL); days post vaccination (dpv); days post infection (dpi); codon-pair deoptimization (CPD); fluorescent focus neutralization (FFN); 50% tissue culture infectious dose (TCID50); cell-mediated immune (CMI); enzyme-linked immunosorbent spot (ELISPOT assay); peripheral blood mononuclear cells (PBMCs); tumor necrosis factor alpha (TNF-α); high-pathogenic (HP); low-pathogenic (LP); secreting cells (SCs).
